# Somatic complaints in children and adolescents with social anxiety disorder

**DOI:** 10.1007/s40211-018-0288-8

**Published:** 2018-09-14

**Authors:** Petra Sackl-Pammer, Zeliha Özlü-Erkilic, Rebecca Jahn, Andreas Karwautz, Eva Pollak, Susanne Ohmann, Türkan Akkaya-Kalayci

**Affiliations:** 1 Department of Child and Adolescent Psychiatry, Medical University of Vienna, Währinger Gürtel 18–20, 1090 Vienna, Austria; 2 Outpatient Clinic of Transcultural Psychiatry and Migration Induced Disorders in Childhood and Adolescence, Department of Child and Adolescent Psychiatry, Medical University of Vienna, Währinger Gürtel 18–20, 1090 Vienna, Austria; 3 Department for Psychiatry and Psychotherapy, Clinical Division of Social Psychiatry, Medical University of Vienna, Währinger Gürtel 18–20, 1090 Vienna, Austria

**Keywords:** Social anxiety disorder, Somatic symptoms, Children, Adolescents, Social phobia, Soziale Angststörung, Somatische Symptome, Kinder, Jugendliche, Soziale Phobie

## Abstract

**Background:**

Associations of social anxiety disorder (SAD) with various somatic symptoms have been already reported in the literature several times. The present study investigated somatic complaints in children and adolescents with SAD compared to controls and evaluated the relationship between social anxiety and somatic symptom severity.

**Methods:**

Thirty children and adolescents with SAD were compared with 36 healthy age-matched controls. Self-reported fears were assessed using the Phobiefragebogen für Kinder und Jugendliche (PHOKI); emotional and behavioral problems were assessed using the Child Behavior Checklist (CBCL/4-18); and the Gießener Beschwerdebogen für Kinder und Jugendliche (GBB-KJ) was used to assess 59 somatic symptoms.

**Results:**

Parents and youth with SAD reported higher somatic symptom severity compared to controls. Youth with SAD more frequently reported stomach pain, circulatory complaints, and fatigue than controls. Specific group differences between SAD and control youth were found for the following single somatic symptoms: faintness, quickly exhausted, sensation of heat, stomachache, nausea, dizziness, and sudden heart complaints. Parents of girls with SAD reported higher somatic symptom severity than parents of boys with SAD.

**Conclusions:**

The results demonstrated a significant positive association between somatic symptoms and social anxiety in youth. The results of the present study can help to develop improved screening measurements, which increase the proportion of children and adolescents with SAD receiving proper treatment.

## Zusammenfassung

**Grundlagen:**

In der Literatur wurden bereits mehrfach die Assoziationen einer sozialen Angststörung (SAS) mit verschiedenen somatischen Symptomen beschrieben. Die vorliegende Studie untersuchte somatische Beschwerden bei Kindern und Jugendlichen mit SAS im Vergleich zu Kontrollpersonen und bewertete die Beziehung zwischen sozialer Angst und dem Schweregrad der somatischen Symptome.

**Methodik:**

Dreißig Kinder und Jugendliche mit SAS wurden mit 36 gesunden altersgematchten Kontrollpersonen verglichen. Selbstberichtete Ängste wurden mit dem Phobiefragebogen für Kinder und Jugendliche (PHOKI) bewertet; emotionale und Verhaltensprobleme wurden anhand der Child Behavior Checklist (CBCL/4-18) beurteilt; und der Gießener Beschwerdebogen für Kinder und Jugendliche (GBB-KJ) wurde verwendet, um 59 somatische Symptome zu erfassen.

**Ergebnisse:**

Eltern und Kinder bzw. Jugendliche mit SAS berichteten über einen höheren Schweregrad der somatischen Symptome im Vergleich zu Kontrollpersonen. Kinder und Jugendliche mit SAS berichteten häufiger über Magenschmerzen, Kreislaufbeschwerden und anhaltende Müdigkeit als Kontrollpersonen. Spezifische Gruppenunterschiede zwischen Kindern bzw. Jugendlichen mit SAS und den Kontrollpersonen wurden für folgende einzelne somatische Symptome gefunden: Ohnmachtsgefühle, Erschöpfungssyndrom, Hitzegefühl, Bauchschmerzen, Übelkeit, Schwindel und plötzliche Herzbeschwerden. Eltern von weiblichen Studienteilnehmern mit SAS berichteten über eine höhere somatische Symptomschwere als Eltern von männlichen Studienteilnehmern mit einer entsprechenden SAS.

**Schlussfolgerungen:**

Die Ergebnisse zeigten, dass bei Kindern und Jugendlichen eine signifikante positive Assoziation zwischen somatischen Symptomen und sozialer Angst besteht. Die Ergebnisse der vorliegenden Studie können dazu beitragen, verbesserte Screening-Messungen zu entwickeln, die den Anteil von Kindern und Jugendlichen mit SAS schneller erfassen, um ihnen zeitnaher eine angemessene multiprofessionelle Behandlung zu ermöglichen.

## Introduction

Social anxiety disorder (SAD) is a very common mental health disorder [1] that typically begins in childhood or adolescence [2, 3] with highest incidence rates between the ages of 10 and 19 years [4].

SAD can be associated with various impairments, with the effects on social [5] and academic [5, 6] lives being highly detrimental for children. This expresses itself in higher scores on a loneliness scale and having fewer friends than their age-matched peers [5]. A negative attitude towards school and therefore irregular attendance and high drop-out rates are also typical for children with SAD [5, 6]. Adult SAD patients show impairment in their social and professional role functioning too [7–9]. Even reduced quality of life is often reported [1, 10].

An association of SAD with other mental health disorders, especially anxiety [2, 8] and affective disorders [8, 9, 11], is quite common and comorbidity rates of up to 60% have been reported [9, 12]. Furthermore, SAD was identified as a risk factor for alcohol and cannabis dependency [13].

Somatic complaints are also highly associated with anxiety disorders in general [14, 15] as well as with social anxiety disorder in particular [15, 16].

In studies involving samples of adolescent clinical patients with anxiety disorders, more than 90% of the participants reported suffering from at least one somatic symptom [15, 17–19]. Additionally, multiple studies showed a positive correlation between somatic symptom severity and the severity of the anxiety disorder as well as the degree of the general impairment [17, 19, 20].

Due to their temporal appearance, Janssens et al. [21] was able to identify anxiety disorders and depressive episodes as risk factors for the development of somatic complaints using data of the TRAILS study (Tracking Adolescents’ Individual Life Survey).

In the study of Ginsburg et al. [19] the somatic symptoms of children and adolescents with either social anxiety disorder, separation anxiety disorder or generalized anxiety disorder differed only slightly. So unrest and stomach pain were the most common symptoms for all three diagnoses while blushing was the third most often reported symptom in children and adolescents with SAD. On the other hand, children with SAD in the study of Hofflich et al. [15] named feeling tense and feeling strange or unreal as their most prevalent symptoms. By means of two multiple linear regression models, May et al. [20] could establish nausea and muscle tension as significant predictors for the interaction type of SAD disorder and together they accounted for 72% of the variance in interaction anxiety. Similarly, accelerated heartbeat and chest pain accounted for 64% of the variance in performance anxiety.

Blushing is also an often reported symptom of SAD and many patients are even afraid of this symptom because it can be noticed by other people. However, Gerlach et al. [22] found that only one out of three tasks caused adult patients with SAD to blush more than healthy controls, even though they reported blushing more in every task. Therefore, the authors supposed that there are differences in body perception between people with SAD and healthy controls. There are results indicating that an increased body perception correlates positively with somatic symptom severity [23].

Children and adolescents with SAD and comorbid depression had a higher number of symptoms and somatic symptom severity than those with just SAD alone [15, 17].

The influence of gender and age on number and severity of somatic symptoms is still a matter of controversial discussion. While some studies could not find an association between age, gender and somatic symptom severity [15, 24] in children and adolescents with anxiety disorders, others reported an increased number of somatic symptoms in older [17, 19] or female participants [25].

There are multiple ways in which somatic complaints can affect children and adolescents with SAD negatively. Apart from the negative effects of anxiety disorders on academic performance (as described above), Hughes et al. [24] showed that the level of self-reported and parent-reported somatic symptom severity were significant predictors of academic performance independently of the presence of anxiety symptoms. Children and adolescents with SAD and a higher level of somatic symptoms avoided fear-inducing situations more often and familiar relationships were more problematic [19]. In the first place most children and adolescents with SAD see a general physician or a paediatrician and sometimes they do not receive any treatment by a mental health care professional at all. This could be also due to the presence of somatic symptoms which are often most prominent in children with SAD and therefore being the reason for the medical consultation [26]. That is why Masia Warner et al. [27] argued that closer cooperation between general physicians, paediatricians and mental health care professionals and improved screening measurements are needed to ensure a prompt and appropriate treatment for children and adolescents with anxiety disorders. According to Campo [25] improved screening measurements of psychopathology in primary care could facilitate the access to an adequate treatment of affected children and their families.

There are inconclusive results about the efficacy of well-established therapy strategies for childhood anxiety disorders in reducing somatic symptoms. In one study the treatment with SSRI (Selective serotonin reuptake inhibitor) was superior to a placebo pill condition in reducing somatic symptoms [19]. In another study, there was no significant difference in the reduction of somatic symptoms between active treatment strategies (CBT [Cognitive behavioral therapy], SSRI or combination) and the placebo pill condition [17]. Therefore, a new psychosocial treatment concept, TAPS (Treatment of Anxiety and Physical Symptoms) was developed concentrating on both the anxiety and the somatic symptoms. In a pilot study of Masia Warner et al. [27] first indications of the efficacy of the TAPS program could be found.

Most of the previous studies [28, 29] focusing on SAD did not include a clinical and a control group, in order to compare patients with and without SAD diagnosis. Moreover, former studies [28–31] frequently included adult patients by analysing SAD. In the present study, we exclusively compared children and adolescents with and without SAD, in order to analyse the relationship between somatic symptom severity and social anxiety. These methodological features add new aspects and improve the knowledge in the field of SAD.

### Aims of the study

The current study was conducted to improve the knowledge about somatic complaints and the relationship between somatic symptom severity and social anxiety in children and adolescents with SAD. Associations of SAD with various somatic symptoms have been reported in the literature before. It was therefore assumed that participants with SAD suffer from somatic symptoms in general as well as from stomach pain and circulatory complaints in particular more often compared to a healthy control group (CON). A positive association between somatic symptom severity and social anxiety was presumed, too. Additionally, an exploratory investigation was conducted comparing the clinical sample (CLIN) with CON regarding single somatic symptoms. Potential influences of gender and age were also investigated.

## Methods

### Participants

The clinical sample (CLIN) consisted of 30 patients (11;0–16;11 years, in- and outpatient). All of them fulfilled the ICD-10 diagnostic criteria for SAD. A total of 36 healthy age-mates served as CON. Additionally, at least one parent of each participant took part in the study. Exclusion criteria for both groups were (a) an IQ below 70 and (b) insufficient knowledge of the German language.

Children and adolescents, who were diagnosed with social phobia according to ICD-10 (F-40.1) by a specialist, were recruited to the outpatient, semi-inpatient and inpatient departments. Either the diagnosis of social phobia was detected by a specialist according to the ICD-10 criteria (F-40.1) or it already existed at the time of examination, or else, there was a preliminary tentative diagnosis, which was subsequently followed diagnostically and led to it being included in the study after hedging the diagnosis.

Among the participants of the control group, two questionnaires, Phobiefragebogen für Kinder und Jugendliche (PHOKI) and the Child Behavior Checklist (CBCL), were used as a screening procedure. The cut-off for CBCL is by >70 (values above that would count as clinically apparent). On the other hand, the cut-off in PHOKI, is the stanine value of over 7, which should count as clinically apparent. Only children and adolescents who had no history of explorable clinical psychopathology, who never had psychiatric treatment, and who currently took no medication, and were below the above-mentioned cut-off criteria in the two questionnaires were included in the control group.

The gender distribution resulted from clinical routine; during the study period slightly fewer female patients, with the diagnosis of social phobia according to ICD-10 criteria, presented at our clinic, as compared to boys. The control group was recruited from youth clubs in Vienna and due to motivational reasons, more girls decided to volunteer than boys. Because of this, participants were just matched by age, but not additionally by sex.

### Measures

#### Demographic characteristics

To ensure comparability between CLIN and CON various demographic variables were collected, such as age of parents, highest level of education of parents, family status (parents living together/parents are separated), number of siblings, housing conditions.

#### Fears

Various self-reported fears such as school phobia, separation anxiety or social anxiety were assessed using the standardized questionnaire PHOKI (Phobiefragebogen für Kinder und Jugendliche; [32]). Children and Adolescents of CON which scored 7 or higher (stanine) on the subscale *social anxiety *were excluded.

#### Parents rating

At least one parent of each participant completed the Child Behavior Checklist (CBCL/4-18; [33]) which assesses internalizing and externalizing emotional and behavioural problems of their children. In order to ensure mental health of the CON participants those with scores above average were excluded.

#### Somatic complaints

To assess the presence of somatic symptoms, the standardized questionnaire GBB-KJ (Gießener Beschwerdebogen für Kinder und Jugendliche; [34]) was used. Participants rated the frequency of 59 somatic symptoms on a 5-point scale from “never” to “permanent”, resulting in a total value, which can be interpreted as general somatic symptom severity, and five subscales, namely stomach complaints, circulatory complaints, fatigue, limb pain and cold complaints.

Questions about various symptoms by CBCL refer to the last 6 months. According to the manual, PHOKI and the GBB have no time specification but refer to the current examination time.

### Statistical analyses

All statistical analyses were computed with SPSS. In order to measure group differences between CLIN and CON, t‑tests were conducted provided the assumptions were met. If this was not the case non-parametric tests like the Mann–Whitney U test were used. In general, a significance level α of 0.05 was applied. To correct for multiple testing Bonferroni correction was performed, the new significance levels will be given in the respective results section.

Missing data were handled according to the manuals of the respective questionnaires. Missing single items were extrapolated by the sum-score of a scale. If more items were missing in one scale, the participant would be excluded from all analyses regarding this questionnaire. Data of the children were, apart from missing single items, complete. Parents’ rating of 3 children of the CLIN was missing, therefore the number of cases in the analysis of the CBCL data is reduced.

The present study has been approved by the Ethics Committee of the Medical University of Vienna (EK-Nummer: 1693/2012).

## Results

### Demographics

In total, 66 children and adolescents (aged 11;0 to 16;11 years) were included in the current study. The clinical group (CLIN) consisted of 30 participants (14 girls, 16 boys) with an average age of 13.63 years, while CON consisted of 36 participants (25 girls, 11 boys) with an average age of 13.39 years. No significant group differences could be found regarding the age of participants (*z* = 0.07, *p* = 0.500), the gender of the participants χ^2^ (1, *N* = 66) = 3.51, *p* = 0.061, the age of their mothers (*z* = 1.09, *p* = 0.275), the number of siblings, χ^2^ (2, *N* = 59) = 3.43, *p* = 0.180, the highest level of education of their mothers, χ^2^ (2, *N* = 60) = 1.03, *p* = 0.599, or their fathers, χ^2^ (2, *N* = 55) = 4.03, *p* = 0.134.

There were however significant group differences regarding the age of the participants’ fathers (*z* = 2.57, *p* = 0.010) with fathers of the CLIN group significantly older than those of CON. Significantly more families of CON lived in their own houses whereas families of CLIN lived more often in flats, χ^2^ (1, *N* = 57) = 6.37, *p* = 0.012. Regarding family status (parents living together/parents are separated) there was a significant group difference as well, χ^2^ (1, *N* = 60) = 7.81, *p* = 0.005. More than half of CLIN members’ parents were separated (54%), compared to just 19% of CON.

### Fears

Data of the PHOKI were not normally distributed, therefore the Mann–Whitney U test, a nonparametric test, was used to investigate group differences. After Bonferroni correction the level of significance was set at α = 0.006 (i. e., 0.05/8). There were significant group differences in the total value (*z* = 3.85, *p* < 0.001) as well as in the subscales separation anxiety (*z* = 3.28, *p* = 0.001) and school and performance anxiety (*z* = 4.97, *p* < 0.001), with CLIN scoring significantly higher than CON. Table 1 shows descriptive statistics of the PHOKI for both groups.

**Table 1:** Descriptive statistics of the results of the Phobiefragebogen für Kinder und Jugendliche (PHOKI)

		Total	Dangers & Death	Separation anxiety	Social anxiety	Threatening & scary	Animal phobia	Medical treatments	School & performance anxiety
**CLIN** **(*n*** **=** **30)**	Mean	6.23	5.27	6.07	7.87	5.97	4.97	5.73	7.27
	Median	7.00	5.00	6.00	8.00	6.00	6.00	6.00	8.00
	SD	2.012	1.999	2.100	1.252	2.282	2.442	2.532	1.437
**CON** **(*n*** **=** **36)**	Mean	4.22	4.11	4.22	4.08	5.22	5.00	5.25	4.56
	Median	4.00	4.00	4.00	4.00	5.00	5.00	5.00	4.50
	SD	1.570	1.720	1.742	1.763	2.085	1.836	1.538	2.063
–	*p*-value	**<0.001**	0.029	**0.001**	**<0.001**	0.178	0.788	0.149	**<0.001**

Significant results are bold

*SD* standard deviation, *CLIN* clinical sample, *CON* controls

### Parent rating

There were significant group differences regarding the total value of the CBCL/4-18 which is regarded as a general indicator of mental health problems, *t*(43.66) = 8.58, *p* < 0.001, with CLIN scoring higher than CON. Both groups also differed significantly in both subscales internalizing problems, *t*(41.86) = 9.74, *p* < 0.001, and externalizing problems, *t*(41.74) = 2.03, *p* = 0.049, with CLIN scoring higher than CON. Table 2 contains means and standard deviations for both groups.

**Table 2:** Descriptive statistics of the Child Behavior Checklist (CBCL/4-18). Means and standard deviations of the CBCL/4-18 for both groups (CLIN and CON)

CBCL/4–18 scales		*N*	Mean	SD	*p*-value
Internalizing problems	CON	36	45.69	6,944	**<0.001**
	CLIN	27	68.74	10,719	
Externalizing problems	CON	36	44.92	8,230	**0.049**
	CLIN	27	50.63	12,759	
Total	CON	36	44.64	7,235	**<0.001**
	CLIN	27	64.85	10,513	

Group differences in the parent-rating of somatic symptoms (subscale somatic complaints of the CBCL) were identified using a Mann–Whitney Utest, which was applied due to non-satisfied requirements for conducting a t-test.

Significant results are marked bold

*SD* standard deviation, *CLIN* clinical sample, *CON* controls

There was a significant difference between the parent-rating of CLIN (*Mdn* = 67.00, *Q1* = 55.00, *Q3* = 77.00) and CON (*Mdn* = 50.00, *Q1* = 50.00, *Q3* = 55.00), *z* = 5.01, *p* < 0.001, with parents of CLIN members reporting higher symptom levels.

### Self-reported somatic complaints

**Fig. 1:**
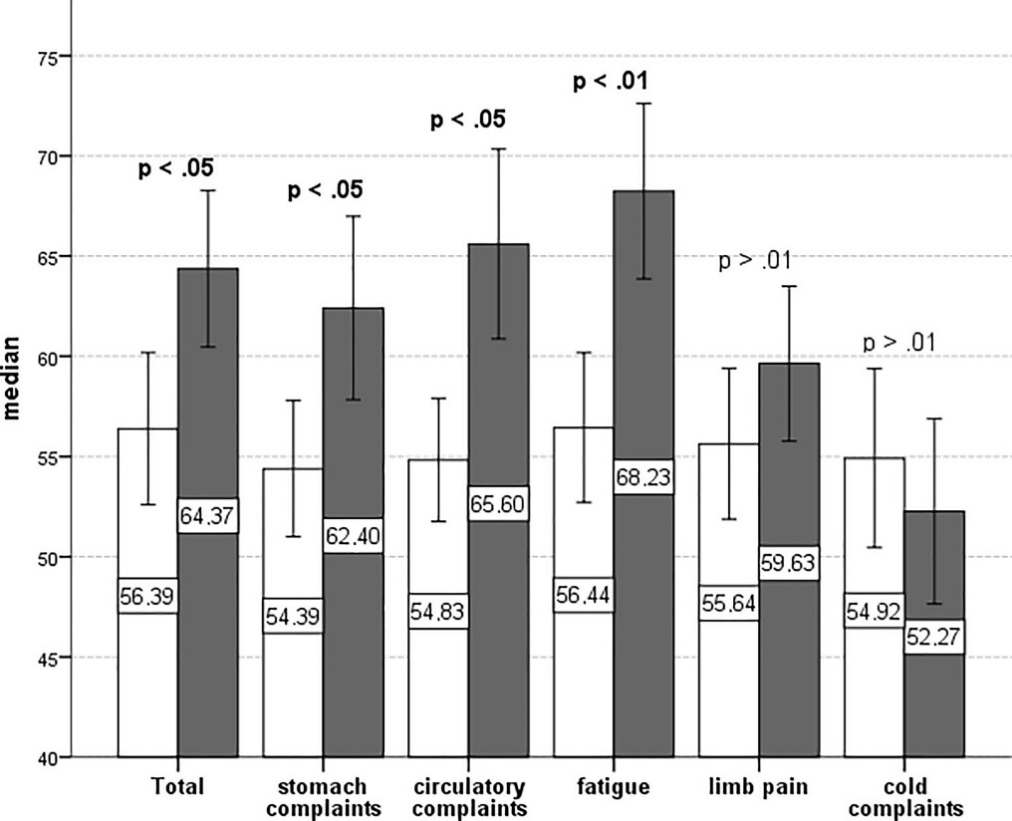
Group comparisons of symptom severity. Means of symptom severity and somatic complaints (subscales of the GBB-KJ) for both groups, CLIN (*n* = 30) and CON (*n* = 36), with error bars marking the 95% CI. Applying Bonferroni correction for the subscales fatigue, limb pain and cold complaints the level of significance was set at α = 0.016. Significant differences are highlighted

In order to compare the two groups (CLIN and CON), Mann–Whitney U tests were conducted because the data was not normally distributed and therefore the requirements for t‑tests were not satisfied. For the subscales *fatigue, limb pain *and *cold complaints *a Bonferroni correction of the level of significance was applied because there were no a priori hypotheses regarding these scales. Fig. 1 illustrates the group differences, with emphasis on significant results.

We were able to confirm the hypothesis that members of CLIN showed greater somatic symptom severity than CON. As assumed, participants of CLIN suffered more from stomach pain and circulatory complaints. There was a significant group difference in the subscale fatigue too.

### Single somatic symptoms

In an explorative analysis group differences of single somatic symptoms were investigated for those 20 items defining the three subscales: fatigue, stomach pain and circulatory complaints. Due to the nonsatisfied requirements of the t‑test, Mann–Whitney U tests were conducted. The level of significance was set at *p* < 0.0025 (0.05/20), applying Bonferroni correction. Table 3 shows symptoms in which the groups significantly differed.

**Table 3:** Group comparisons of somatic symptoms. Group comparisons of single somatic symptoms using Mann–Whitney U tests (only significant results are shown)

	Faintness	Quickly exhausted	Sensation of heat	Stomach ache	Nausea	Dizziness	Sudden heart complaints
Mann–Whitney U	286.500	262.500	306.000	312.500	314.500	253.500	349.500
Wilcoxon W	952.500	928.500	972.000	978.500	980.500	919.500	1,015.500
Z	−3.360	−3.717	−3.137	−3.031	−3.031	−3.916	−3.079
Significance (two-tailed)	0.001	0.000	0.002	0.002	0.002	0.000	0.002

### Association between somatic complaints and social anxiety

In order to investigate the relationship between social anxiety and somatic symptoms (independent of group membership), partial correlations were calculated. Results are shown in Table 4. As assumed, there was a significant positive correlation between social anxiety and somatic symptom severity, controlling for group membership. Additionally, positive correlation between social anxiety and fatigue, circulatory complaints, limb pain, and cold complaints were found, controlling for group membership.

**Table 4:** Partial correlation between social anxiety and somatic complaints. Partial correlation between the subscale social anxiety of the PHOKI („Phobiefragebogen für Kinder und Jugendliche“) and the total value as well as the subscales of the GBB-KJ („Gießener Beschwerdebogen für Kinder und Jugendliche“) controlling for group membership

		GBB-KJ total	Fatigue	Stomach complaints	Circulatory complaints	Limb pain	Cold complaints
PHOKI social anxiety	Correlation	0.517	0.408	0.199	0.605	0.372	0.454
	Significance	0.000	0.001	0.112	0.000	0.002	0.000
	Degrees of freedom	63	63	63	63	63	63

### Gender and age

There was no gender difference in the self-reported somatic symptom severity as well as in the subscales of the GBB-KJ (fatigue, stomach complaints, circulatory complaints, limb pain, and cold complaints). Regarding the parent-rating of somatic symptoms there was a significant gender difference in CLIN (*z* = 2.07, *p* = 0.03) with parents of girls (*mdn* = 75.00, *Q*_*1*_ = 66.00, *Q*_*3*_ = 77.50) reporting higher somatic symptom severity than parents of boys (*mdn* = 65.50, *Q*_*1*_ = 54.00, *Q*_*3*_ = 68.00). No such difference could be identified for CON (*z* = 0.16, *p* = 0.867).

There was no significant relationship between age and somatic symptom severity as well as the subscales of the GBB-KJ.

## Discussion

The aim of the current study was to investigate somatic complaints in children and adolescents with SAD and to examine the relationship between social anxiety and somatic symptom severity in general as well as different somatic complaints in particular. This knowledge could lead to improved screening measurements and therefore increase the proportion of children and adolescents with SAD receiving proper treatment.

In this study, the association between somatic symptoms and social phobia in childhood and adolescence was clearly demonstrated. Referring to these findings, diagnosing in the primary care setting can especially be accelerated in the fields of paediatrics and psychiatry by using the testing methods (CBCL, GBB, PHOKI) adequately at the medical investigation of somatic symptoms. By using this method, children/adolescents would often be spared many long, exhausting, and often painful organic investigations, or these could be significantly minimized. Paediatricians can rapidly do the screening procedure, and upon finding suspicious test results, may start earlier with a close specialist and psychotherapeutic treatment, which can lead to reduction of symptoms and to stabilizing of the social phobia in childhood and adolescence, often just a few weeks/months later.

There was a significant group difference in self-reported and parent-reported somatic symptom severity with children and adolescents in CLIN suffering from a higher somatic symptom severity than those in CON. This finding is in line with previous research [15, 16].

Additionally participants of CLIN reported a significant higher frequency of stomach complaints, circulatory complaints and fatigue.

Significant group differences were also found for the single somatic symptoms faintness, quickly exhausted, sensation of heat, stomach ache, nausea, dizziness and sudden heart complaints.

Stomach pain [19], nausea and accelerated heart beat [20] were the most common reported symptoms in the literature supporting our findings that CLIN suffered more from stomach complaints and circulatory complaints in general as well as from stomach ache, nausea and sudden heart complaints in particular. However, children and adolescents with SAD suffering more from fatigue were not mentioned in the literature. This result is maybe caused by comorbid depressive symptoms in CLIN which epidemiologic studies have shown are likely to co-occur with SAD [8, 9, 11]. Fatigue in turn is one of the cardinal symptoms of depressive disorders [35, 36].

The somatic symptom sensation of heat is comparable with the symptom blushing which is common and often feared in patients with SAD [22].

## Conclusion

Social anxiety was positively associated with somatic symptom severity as well as with fatigue, circulatory complaints, limb pain and cold complaints controlling for group membership. So independent of the presence of a SAD diagnosis and of the potential presence of comorbidities in CLIN a relationship between social anxiety and somatic complaints could be identified. Actually even a broader range of symptom qualities was associated with social anxiety than suggested by group comparisons. These results are in line with other studies finding associations between social anxiety and somatic symptom severity in nonclinical samples [16].

In conclusion, a significant positive relationship between social anxiety and somatic symptom severity could be proven. Nevertheless, this result should be interpreted with caution; as no comorbidities were assessed, it is difficult to deduce if the reported symptoms are caused only by SAD or are (partly) an expression of some other comorbid psychiatric disorder(s). The clinical sample of the current study suffered from a high number and frequency of somatic symptoms which is in line with several other studies. Moreover Hughes et al. [24] established somatic symptom severity as a significant predictor of negative academic performance independently of anxiety symptom severity. So it can be justified to specifically search for somatic symptoms in children and adolescents with SAD and monitor them in the course of therapy. If sufficient symptom reduction cannot be achieved by the means of conventional therapy programs of SAD as it could be shown in some studies [17, 19] it may be helpful to apply a therapy strategy especially developed to reduce SAD and somatic symptoms [27].

### Limitations

The current study has some limitations. The first limitation concerns comorbidity which was not assessed and therefore not controlled for. Epidemiologic studies showed that SAD patients often suffer from additional internalizing disorders which could have influenced our results.

Another limitation was that we did not control for ongoing treatment among the members of the clinical group. The possibility of influences due to medical treatment cannot be ruled out.

Additionally, only subjective data was reported by children and their parents, while no objective measurements of somatic symptoms were done.

Some of the study participants had previous treatments by psychotherapists or specialists in the past, but this has not been systematically documented and is one of the limitations of the present study.

The primary diagnosis “social phobias” according to ICD-10 (F 40.1) was recorded by a specialist and psychologist for the inpatient, day-patient and for acute treatment. While recruiting the patients, care was taken that no long-term medication (longer than 2 weeks) was being taken at the time of the examination. However, among a few patients, acute medication in order to cope with anxiety, was documented. Whether patients were currently undergoing psychotherapy or had previous experience with psychological care was unfortunately not recorded at the time of examination. Regrettably, also mental disorders of the parents were not recorded.

In the present study, the patients as well as the subjects of the control group were investigated individually. The gender distribution resulted from clinical routine; as during the study period slightly fewer female patients, with the diagnosis of social phobia according to ICD-10 criteria, were presented at our clinic, as compared to boys. In contrast, the control group recruited from youth clubs in Vienna, where more girls decided to volunteer than boys. Because of this, participants were not matched by sex. It cannot be precluded whether this biased the results.

### Strength

One of the strengths of this study was the inclusion of a clinical group with a primary diagnosis of SAD confirmed by a mental health professional. There are only a few studies (especially with children and adolescents) that included clinical groups. In the meta-analysis by Aldao et al. [37] for example there was no study that involved a clinical group of children and adolescents.

Another strong point of this study was that in addition to self-reported somatic symptoms, parent rating was taken into account too. Therefore, somatic symptom severity could be studied from different angles.

### Future directions

Future studies are needed to investigate the causal associations between somatic symptom severity and SAD in children and adolescents. In order to compare and specify the disorder-specific somatic complaints profile, other anxiety disorders should be included in the investigation. Furthermore, existing therapy programs of SAD should be evaluated with respect to somatic symptom reduction and new approaches to alleviate somatic symptoms in children and adolescents with anxiety disorders should be evaluated.

## References

[1] Stein MB (2006). An epidemiologic perspective on social anxiety disorder. J Clin Psychiatry.

[2] Burstein M, He JP, Kattan G, Albano AM, Avenevoli S, Merikangas KR (2011). Social phobia and subtypes in the national comorbidity survey-adolescent supplement: prevalence, correlates, and comorbidity. J Am Acad Child Adolesc Psychiatry.

[3] Kessler RC, Angermeyer M, Anthony JC, De Graaf R, Demyttenaere K, Gasquet I (2007). Lifetime prevalence and age-of-onset distributions of mental disorders in the World Health Organization’s World Mental Health Survey Initiative. World Psychiatry.

[4] Beesdo K, Bittner A, Pine DS, Stein MB, Hofler M, Lieb R (2007). Incidence of social anxiety disorder and the consistent risk for secondary depression in the first three decades of life. Arch Gen Psychiatry.

[5] Beidel DC, Turner SM, Morris TL (1999). Psychopathology of childhood social phobia. J Am Acad Child Adolesc Psychiatry.

[6] Van Ameringen M, Mancini C, Farvolden P (2003). The impact of anxiety disorders on educational achievement. J Anxiety Disord.

[7] Aderka IM, Hofmann SG, Nickerson A, Hermesh H, Gilboa-Schechtman E, Marom S (2012). Functional impairment in social anxiety disorder. J Anxiety Disord.

[8] Kroenke K, Spitzer RL, Williams JB, Monahan PO, Lowe B (2007). Anxiety disorders in primary care: prevalence, impairment, comorbidity, and detection. Ann Intern Med.

[9] Ruscio AM, Brown TA, Chiu WT, Sareen J, Stein MB, Kessler RC (2008). Social fears and social phobia in the USA: results from the National Comorbidity Survey Replication. Psychol Med.

[10] Mendlowicz MV, Stein MB (2000). Quality of life in individuals with anxiety disorders. Am J Psychiatry.

[11] Pohlmann K, Dobbel S, Loffler S, Israel M, Joraschky P (2009). Social phobia—the blind spot: infrequently diagnosed, highly complex, and a predictor for unfavourable therapy outcomes?. Z Psychosom Med Psychother.

[12] Leyfer O, Gallo KP, Cooper-Vince C, Pincus DB (2013). Patterns and predictors of comorbidity of DSM-IV anxiety disorders in a clinical sample of children and adolescents. J Anxiety Disord.

[13] Buckner JD, Schmidt NB, Lang AR, Small JW, Schlauch RC, Lewinsohn PM (2008). Specificity of social anxiety disorder as a risk factor for alcohol and cannabis dependence. J Psychiatr Res.

[14] Beidel DC, Christ MG, Long PJ (1991). Somatic complaints in anxious children. J Abnorm Child Psychol.

[15] Hofflich SA, Hughes AA, Kendall PC (2006). Somatic complaints and childhood anxiety disorders. Int J Clin Health Psychol.

[16] Jellesma FC, Rieffe C, Terwogt MM (2008). My peers, my friend, and I: peer interactions and somatic complaints in boys and girls. Soc Sci Med.

[17] Crawley SA, Caporino NE, Birmaher B, Ginsburg G, Piacentini J, Albano AM (2014). Somatic complaints in anxious youth. Child Psychiatry Hum Dev.

[18] Ferdinand RF, Verhulst FC (1995). Psychopathology from adolescence into young adulthood: an 8‑year follow-up study. Am J Psychiatry.

[19] Ginsburg GS, Riddle MA, Davies M (2006). Somatic symptoms in children and adolescents with anxiety disorders. J Am Acad Child Adolesc Psychiatry.

[20] May AC, Rudy BM, Davis TE, Jenkins WS, Reuther ET, Whiting SE (2014). Somatic symptoms in those with performance and interaction anxiety. J Health Psychol.

[21] Janssens KA, Rosmalen JG, Ormel J, van Oort FV, Oldehinkel AJ (2010). Anxiety and depression are risk factors rather than consequences of functional somatic symptoms in a general population of adolescents: the TRAILS study. J Child Psychol Psychiatry.

[22] Gerlach AL, Wilhelm FH, Gruber K, Roth WT (2001). Blushing and physiological arousability in social phobia. J Abnorm Psychol.

[23] Villanueva L, Gorriz AB, Prado-Gasco V, Gonzalez R (2015). The role of emotion awareness and mood: somatic complaints and social adjustment in late childhood. Psychol Health Med.

[24] Hughes AA, Lourea-Waddell B, Kendall PC (2008). Somatic complaints in children with anxiety disorders and their unique prediction of poorer academic performance. Child Psychiatry Hum Dev.

[25] Campo JV (2012). Annual research review: functional somatic symptoms and associated anxiety and depression—developmental psychopathology in pediatric practice. J Child Psychol Psychiatry.

[26] Ramsawh HJ, Chavira DA, Stein MB (2010). Burden of anxiety disorders in pediatric medical settings: prevalence, phenomenology, and a research agenda. Arch Pediatr Adolesc Med.

[27] Masia Warner C, Reigada LC, Fisher PH, Saborsky AL, Benkov KJ (2009). CBT for anxiety and associated somatic complaints in pediatric medical settings: an open pilot study. J Clin Psychol Med Settings.

[28] Alden LE, Buhr K, Robichaud M, Trew JL, Plasencia ML (2018). Treatment of social approach processes in adults with social anxiety disorder. J Consult Clin Psychol.

[29] Strege MV, Swain D, Bochicchio L, Valdespino A, Richey JA (2018). A pilot study of the effects of mindfulness-based cognitive therapy on positive affect and social anxiety symptoms. Front Psychol.

[30] Bielak T, Moscovitch DA, Waechter S (2018). Out of my league: appraisals of anxiety and confidence in others by individuals with and without social anxiety disorder. J Anxiety Disord.

[31] Wermes R, Lincoln TM, Helbig-Lang S (2018). Attentional biases to threat in social anxiety disorder: time to focus our attention elsewhere?. Anxiety Stress Coping.

[32] Döpfner M, Schnabel M, Goletz H, Ollendick H (2006). Phobiefragebogen für Kinder und Jugendliche (PHOKI).

[33] Arbeitsgruppe Deutsche Child Behavior Checklist (1998). Elternfragebogen über das Verhalten von Kindern und Jugendlichen: Deutsche Bearbeitung der Child Behavior Checklist (CBCL/4-18), Einführung und Anleitung zur Handauswertung.

[34] Brähler E (1992). Gießener Beschwerdebogen für Kinder und Jugendliche (GBB-KJ).

[35] Sass H, Wittchen HU, Zaudig M, Houben I (2003). Diagnostisches und Statistisches Manual Psychischer Störungen DSM-IV-TR: Textrevision.

[36] Schulte-Markwort E, Remschmidt H (2011). Internationale Klassifikation psychischer Störungen: ICD-10 Kapitel V (F). Klinisch-diagnostische Leitlinien.

[37] Aldao A, Nolen-Hoeksema S, Schweizer S (2010). Emotion-regulation strategies across psychopathology: a meta-analytic review. Clin Psychol Rev.

